# Supplementary material for: Exploiting attP landing sites and gypsy retrovirus insulators to identify and study viral suppressors of RNA silencing
Source: bioRxiv. 2025 Feb 14:2025.02.12.637972. Preprint. [Version 1] doi: 10.1101/2025.02.12.637972 (PMC11844480; doi:10.1101/2025.02.12.637972)
Supplement: 1 — Figure S1. Map of UAS expression vector. Vector with FHV-B2 (black) and flanking gypsy elements (maroon) is shown here as an example. The full sequence of this construct has been deposited in the GenBank database under the accession number OR769027. Figure S2. Confirmation of sequence accuracy for UAS-FHV-B2 vector. The FHV-B2 reference sequence was obtained from GenBank, accession no. NC_004146.1. Figure S3. Western blot confirming expression of FHV-B2 protein. Lane 1, MBP-tagged B2 protein expressed from bacteria (positive control). Lanes 2 and 3, Expression of FHV-B2 in GMR-whiteIR; GMR-GAL4; UAS-FHV (chromosome 2; VK1 or X chromosome; attP18) flies. Lane 4, EGFP-expressed from GMR-whiteIR; GMR-GAL4; UAS-GFP (VK-1) flies (negative control). Anti-β-Actin was used to show equal loading. [file NIHPP2025.02.12.637972V1-supplement-1.pdf]

bioRxiv preprint doi: https://doi.org/10.1101/2025.02.12.637972; this version posted February 14, 2025. The copyright holder for this preprint (which was not certified by peer review) is the author/funder, who has granted bioRxiv a license to display the preprint in perpetuity. It is made available under aCC-BY 4.0 International license.

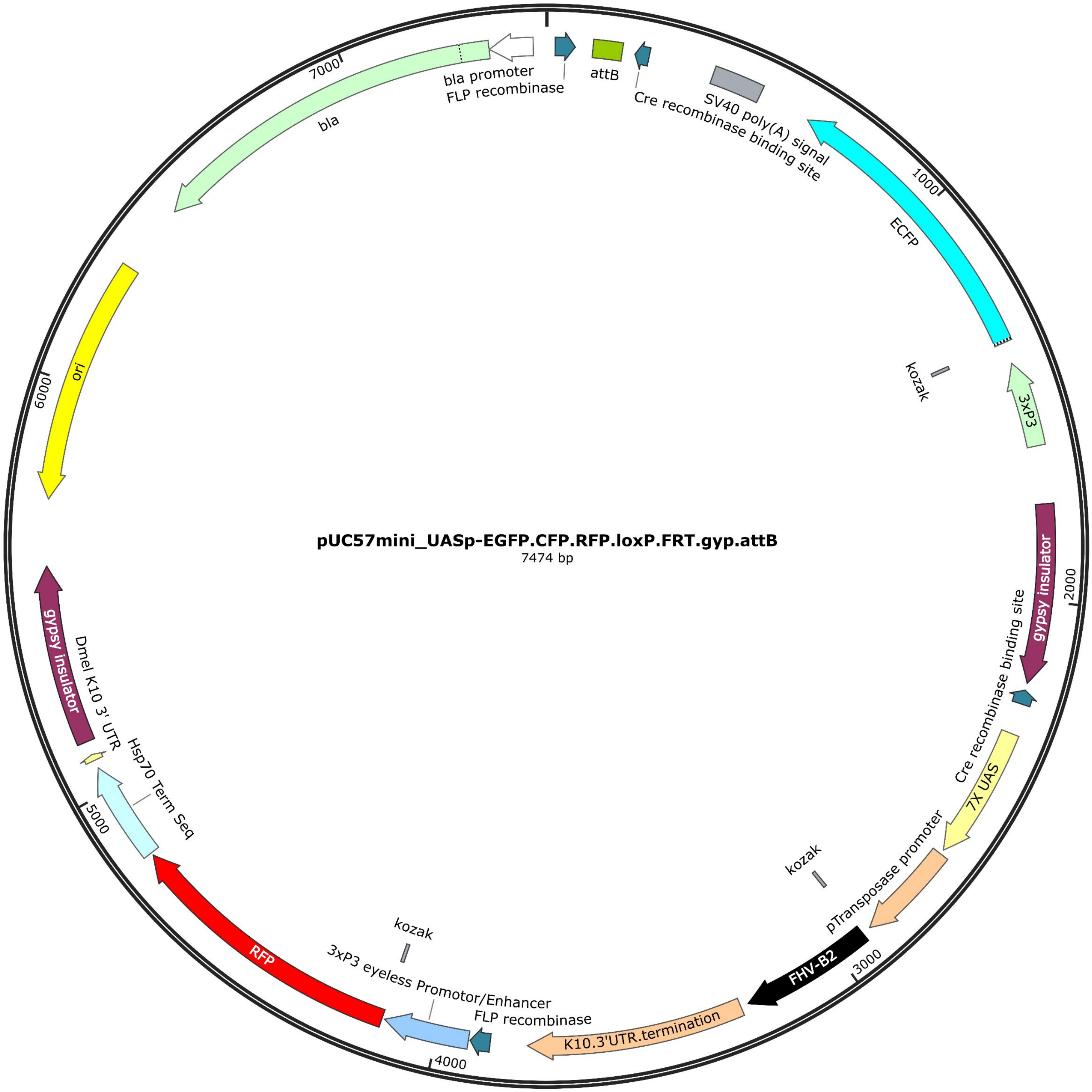

Figure S1

bioRxiv preprint doi: https://doi.org/10.1101/2025.02.12.637972; this version posted February 14, 2025. The copyright holder for this preprint (which was not certified by peer review) is the author/funder, who has granted bioRxiv a license to display the preprint in perpetuity. It is made available under aCC-BY 4.0 International license.

```
FHV-B2_NC_004146.1      ATGCCAAGCAAACTCGCGCTAATCCAGGAACTTCCCGACCGCATTCAAACGGCGGTGGAA    60
FHV-B2_pUAS             ATGCCAAGCAAACTCGCGCTAATCCAGGAACTTCCCGACCGCATTCAAACGGCGGTGGAA    60
FHV-B2_Chr-2            ATGCCAAGCAAACTCGCGCTAATCCAGGAACTTCCCGACCGCATTCAAACGGCGGTGGAA    60
FHV-B2_Chr-X            ATGCCAAGCAAACTCGCGCTAATCCAGGAACTTCCCGACCGCATTCAAACGGCGGTGGAA    60
                        ************************************************************

FHV-B2_NC_004146.1      GCAGCCATGGGAATGAGCTACCAAGACGCACCGAACAACGTGCGCAGGGACCTCGACAAC    120
FHV-B2_pUAS             GCAGCCATGGGAATGAGCTACCAAGACGCACCGAACAACGTGCGCAGGGACCTCGACAAC    120
FHV-B2_Chr-2            GCAGCCATGGGAATGAGCTACCAAGACGCACCGAACAACGTGCGCAGGGACCTCGACAAC    120
FHV-B2_Chr-X            GCAGCCATGGGAATGAGCTACCAAGACGCACCGAACAACGTGCGCAGGGACCTCGACAAC    120
                        ************************************************************

FHV-B2_NC_004146.1      CTGCACGCTTGCCTAAACAAGGCAAAACTAACGGTAAGTCGGATGGTAACATCACTGCTG    180
FHV-B2_pUAS             CTGCACGCTTGCCTAAACAAGGCAAAACTAACGGTAAGTCGGATGGTAACATCACTGCTG    180
FHV-B2_Chr-2            CTGCACGCTTGCCTAAACAAGGCAAAACTAACGGTAAGTCGGATGGTAACATCACTGCTG    180
FHV-B2_Chr-X            CTGCACGCTTGCCTAAACAAGGCAAAACTAACGGTAAGTCGGATGGTAACATCACTGCTG    180
                        ************************************************************

FHV-B2_NC_004146.1      GAGAAACCCAGCGTGGTGGCATACCTAGAGGGAAAGGCCCCCGAGGAGGCAAAACCAACA    240
FHV-B2_pUAS             GAGAAACCCAGCGTGGTGGCATACCTAGAGGGAAAGGCCCCCGAGGAGGCAAAACCAACA    240
FHV-B2_Chr-2            GAGAAACCCAGCGTGGTGGCATACCTAGAGGGAAAGGCCCCCGAGGAGGCAAAACCAACA    240
FHV-B2_Chr-X            GAGAAACCCAGCGTGGTGGCATACCTAGAGGGAAAGGCCCCCGAGGAGGCAAAACCAACA    240
                        ************************************************************

FHV-B2_NC_004146.1      CTCGAAGAACGCCTCCGAAAGCTGGAGCTCAGCCACAGCCTTCCAACAACCGGAAGTGAC    300
FHV-B2_pUAS             CTCGAAGAACGCCTCCGAAAGCTGGAGCTCAGCCACAGCCTTCCAACAACCGGAAGTGAC    300
FHV-B2_Chr-2            CTCGAAGAACGCCTCCGAAAGCTGGAGCTCAGCCACAGCCTTCCAACAACCGGAAGTGAC    300
FHV-B2_Chr-X            CTCGAAGAACGCCTCCGAAAGCTGGAGCTCAGCCACAGCCTTCCAACAACCGGAAGTGAC    300
                        ************************************************************

FHV-B2_NC_004146.1      CCCCCACCCGCAAAACTGTAG   321
FHV-B2_pUAS             CCCCCACCCGCAAAACTGTAG   321
FHV-B2_Chr-2            CCCCCACCCGCAAAACTGTAG   321
FHV-B2_Chr-X            CCCCCACCCGCAAAACTGTAG   321
                        *********************
```

Figure S2

bioRxiv preprint doi: https://doi.org/10.1101/2025.02.12.637972; this version posted February 14, 2025. The copyright holder for this preprint (which was not certified by peer review) is the author/funder, who has granted bioRxiv a license to display the preprint in perpetuity. It is made available under aCC-BY 4.0 International license.

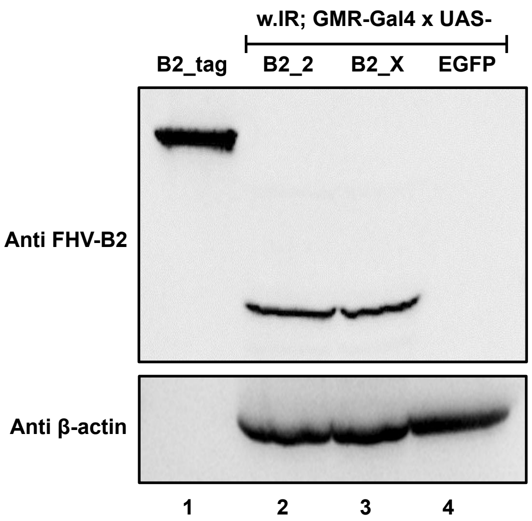

Figure S3
